# The Pervert Wilde

**Published:** 1895-04

**Authors:** 


THE PERVERT, WILDE.—Revelations in London, in connection with the arrest and imprisonment of Oscar Wilde for a "nameless crime," have shed a light, the phosphorent glow rather, that emanates from a charnel house, upon the man's character; and in that light we can now understand the warp of his intellectual nature. The perverted sexual sense has dominated his life, morally and intellectually. We now know him to be a moral monster, and we have always regarded him as an intellectual freak. We can read a deeper meaning now between the thoroughly enough disgusting lines of his salacious novels, and every sense of decency revolts at the suggestions therein brazenly presented, while a blush mantles the reader's cheek. Like some huge, filthy beast, wallowing in the spring whence flows a crystal stream where thousands drink, he has polluted the current of modern light literature; muddied the stream; aye—poisoned it! He is, and has been, an enemy to society, and there is no knowing the amount of harm he has done,—*not* alone in his writings—but by his beastial practices. He has prostituted youths—*boys*—to his depraved, perverted lust, and naturally has made criminals of them; for, what pride, sense of honor, self-respect, or respect for God or man, could such boy ever have, having been so degraded by this beast in human form?

Reformers may prate, and societies for moral purity may resolve; but there is but one remedy for the evil, and that is,—

CASTRATION. In the interest of morality, of humanity, of civilization; in the interest of the rising generations, and for the protection of youth from the accursed perverts of the leisure class of luxurious debauchees to which Wilde and Douglas belong—it is demanded; and Oscar Wilde should be the first example. "Nameless crimes"? Why should a civilization that will tolerate such crime, blush at calling it by its proper name? It is a false modesty. The evil exists. We can not shut our eyes to it, however gladly we would do so. The issue must be met; the evil eradicated.

Laws are made to meet crimes as they are developed in the progress of civilization. There is yet no law for these crimes against nature. In America, however revoting and abominable the crime, the criminal can only be indicted for a misdemeanor, and fined ten or twenty dollars. The statute should be changed, and each crime defined, and classified, and a penalty attached to fit the crime. There is but one penalty for beastiality, pederasty and the like abominations, and that is castration. Imagine the moral effect upon the British aristocracy—the debauchees of his class—that public castration of the beast—Oscar Wilde—would have! It ought to be done!—Justice—poetic justice cries aloud for his damned—thrice damnable appendages.—Upon the altar of morality, in the best interests of an outraged society—as an atonement, they should be offered up for the preservation of virtue and the protection of those youths whose hard lot, no doubt—want—cruel want,—hunger—starvation—drive them to submit to degradation a thousand times worse than death, in the desperate struggle for existence which their cruel environment has entailed. Who does not pity the boy—who can sufficiently execrate or denounce the vile parody on man who would thus gratify his beastly propensity! *Hanging* is too good for him. Let him be castrated and branded—"this is the man whom Lord Queensbury denounced to the police as a *criminal against nature*, the vilest known to even that body."

